# Changing medical education scenario: a wakeup call for reforms in Anatomy Act

**DOI:** 10.1186/s12910-020-00507-0

**Published:** 2020-07-25

**Authors:** Rekha Lalwani, Sheetal Kotgirwar, Sunita Arvind Athavale

**Affiliations:** Department of Anatomy, All India Institute of Medical Sciences, Bhopal, 462020 India

**Keywords:** Cadaver, Dissection, Medical education, Southeast Asia

## Abstract

**Background:**

Anatomy Act provides legal ambit to medical educationists for the acquisition of cadavers. The changing medical education scenario, socio-demographic change, and ethical concerns have necessitated an urgent review of its legal and ethical framework. Suitable amendments addressing the current disparities and deficiencies are long overdue.

**Methods:**

Anatomy Act in India is a state Act, which ensures the provision of human bodies for medical education and research.

The methodology included three components namely:

- Comparison of various Anatomy Acts clause by clause,
- Feedback from anatomists, and
- Formulation of comprehensive model Anatomy Act.

**Results:**

Various Acts studied showed discrepancies in the purpose of the Act, roles and duties of stakeholders, regulation for body donation, the procedure to handle unclaimed bodies, disposal of dissected bodies, etc. No Act defines a donor and neither addresses the issue of transport of anatomical material. Only ten states have a clause for body donation. Acts of only six states have been amended over the last 50 years. Three states denied having an Act. The whole exercise of review of Acts, extensive feedback received from end-users, and taking into account global good practices, culminated in drafting a comprehensive model Anatomy Act founded on ethical principles.

**Conclusion:**

India, with the largest number of medical colleges, is not only at the forefront but also a hub of medical education in the Southeast Asia region. Legal reform can be a torchbearer to promote ethical and transparent practices for obtaining cadavers for other countries of the region with similar socio-demography and shall also motivate anatomic fraternity across the globe for critical analysis of their respective Anatomy Acts.

## Background

Anatomy, the study of the structure of the human body is one of the first, most basic, and yet one of the most important subjects studied by medical students when they start their medical career [1]. Teaching and research in anatomy are mainly based on cadaver dissection. Sound knowledge of anatomy obtained through dissection of the human body is an indispensable part of the education of health care professionals [2]. Anatomy Act ensures the provision of cadavers for education and research under the legal ambit.

The earliest reference for legally acquiring cadavers for dissection dates back to the eighteenth century. The Murder Act, 1752 stipulated that the corpses of the executed murderers could be used for dissection. By the early nineteenth century, when the rise of medical science was occurring, a reduction in the number of executions had caused demand to outstrip supply [3, 4]. To impress the necessity for altering the law upon the government, an Anatomical Society was formed around 1810. The efforts of this body gave rise to the formation of a select committee to report on the question in 1828. During the nineteenth-century, rise in criminal practices for acquiring cadaver, which led to several convictions, further pressed for a Bill. As a result of these efforts the **Anatomy Act, 1832** (2 & 3 Will. IV c.75) was passed in United Kingdom Parliament. It gave legal licenses to teachers and students of anatomy to dissect unclaimed and donated bodies [5]. However, this legislation also became an instrument for the oppression of the poor and destitute. Such practices were curbed after willed body programs/ functional bequest programs came into practice in the latter half of the twentieth-century and donations started to happen for medical education [4].

In India, this initiative, which provided the legal process of acquiring of cadavers for anatomical examination, came in the form of Coroners Act in 1871 which was amended in 1949 as Bombay Anatomy Act, 1949 [6]. In Independent India, the Anatomy Act is a state Act promulgated by the legislature and published in the state government gazette [2]. Every state ought to have one. So, each state formulated its Anatomy Act and some were amended at different times [6–11].

India has seen rapid growth in the number of medical education institutes in the last 25 years, consequently, the number of medical students has increased exponentially leading to the increased requirement of cadavers for medical teaching and research. The major source of cadavers for medical institutes traditionally was from unclaimed bodies. However, a gradual and welcome shift in the source of cadavers, from unclaimed to donated, is also being witnessed in recent times. Availability of unclaimed bodies is on decline probably because of improved communication modalities helping to trace the whereabouts of deceased and legislative compulsion of conduction of post-mortem examination on such bodies rendering them unfit for anatomical study. The awareness of body donation is also on the rise but sociocultural, legal, and trust deficit barriers persist [2, 12].

In the global context, there is an ongoing debate on the use of donated versus unclaimed bodies. Jones and Whitaker (2012) and Champney et al. (2019) argue that the use of unclaimed bodies should completely stop as the practice is not only unethical but also leads to commercialization of dead [13, 14]. Formulation of good practices for body donation by the International Federation of Association of Anatomist (IFAA) [15] addresses the ethical issues and calls for transparency in the process of obtaining anatomic material for teaching and research. Appropriate provisions in the Anatomy Act can provide for a legislative backup to achieve these good practices.

The editorial of Journal of Anatomical Society of India which is an official document of Anatomical Society of India, in 2002, has urged the need of formulation of a draft Act, it states that “As discrepancies exist, betwixt any two or more such state Acts, there is an impending and imminent need to amend, bringing in uniformity. A draft Act should be made for all the states to use as a model of guiding principles for the amendment of the Anatomy Acts. This draft should contain all possible aspects well covered as perceived, after going through the Acts of different states” [16].

Anatomy Acts of various states in India differ on the following counts:

- Purpose of the Act
- Roles and duties of stakeholders
- Regulation of body donation
- Procedure to handle unclaimed bodies
- Maintenance of records
- Handling of disputes
- Penalty
- Disposal of dead bodies.

Certain key areas like transport and transfer of anatomical material and definition of donors are missing in all the Acts. Anatomists as the end-users of the Anatomy Act are expected to lawfully and ethically acquire.

and be in lawful and ethical possession of the anatomical material obtained from dead bodies (bones and cadavers).

While regulatory bodies insist on the availability of anatomical material for teaching and research in medical institutions, however, the available Acts fall short in their provisions for the same [6–11, 17–27]. In contrast, The Transplantation of Human Organs and Tissues Act [28], enacted in 1994, is a comprehensive Act in which the responsibilities and duties of all stakeholders are clearly defined. This Act is uniformly applicable throughout India.

Although anatomists of India express the pressing need for a uniform, comprehensive Act as resolved in general body meeting of the National Conference of Anatomical Society of India, 2013, such an effort still to be initiated [29].

Use of human tissue cannot be dissociated from human feelings, behaviour, values, spiritual beliefs and socio-cultural practices and a delicate balance between the scientific exploration, legality and dignity and rights of the human body is to be maintained for the larger good of the society.

Hence the authors took up this task of formulation of comprehensive draft Anatomy Act with objectives to

- Study the Anatomy Acts of various states and Union Territories of India
- Compare the various provisions of Anatomy Acts of different states and Union Territories of India
- Identify deficiencies and discrepancies in the provisions of the Act in various States and Union Territories of India
- Seek suggestions from end-users regarding addition/ deletion/ modifications in various provisions of the Anatomy Act.
- Formulate a comprehensive Draft Model Anatomy Act founded on ethical principles.

## Methods

The study was initiated after approval from the Research and Review Board (RRB) and the Institutional Human Ethics Committee (IHEC) of our institute. The draft Anatomy Act has been prepared through a review of the Anatomy Acts of various states & Union Territories and wide consultation with anatomy fraternity.

The process included three components namely:

- Review of Anatomy Acts of various states.
- Inputs from the end-users i.e. Anatomy departments of medical colleges.
- Formulation of a draft model Act.

### Review of anatomy acts of various states

The original and amended Anatomy Acts of various states and Union Territories of India were obtained by downloading the Acts through the internet from official websites. In case of unavailability from the internet, Right to Information (RTI) queries were sent to the health secretariats of State and Union Territories of Union of India to get a copy of the Act (Table 1). The various provisions of the Acts were thematically analyzed in a tabulated form. Deficiencies and discrepancies in the Acts of various states were analyzed, considering the changing medical education scenario.

**Table 1:** shows the mode of procurement of the Anatomy Acts of respective states and Union Territories

S.no	Mode of obtaining Anatomy Act	Number	States and Union Territories
1.	Website	11	Maharashtra (MH) [6], Delhi (DL) [7, 30], Goa (GA) [19], Gujarat (GJ) [20], Haryana (HR) [21], Himachal Pradesh (HP) [22], Karnataka (KA) [8],Kerala (KL) [9], Punjab (PB) [25], Chandigarh (CH) [25],Tamil Nadu (TN) [11].
2.	Anatomy Act received in response to RTI	2	Manipur (MN) [24], Sikkim (SK) [26]
3	Act received from stakeholders	5	Assam (AS) [17], Bihar (BR) [18], Madhya Pradesh (MP) [23], Odisha (OD) [10],Uttar Pradesh (UP) [27] .
4.	No Anatomy Act enacted as confirmed by RTI	3	Uttarakhand (UK), Tripura (TR), Jharkhand (JH).
5.	Act denied in response to RTI	1	Jammu and Kashmir(J&K).
6.	No Response or inappropriate response to RTI	14	Andhra Pradesh (AP), Arunachal Pradesh^a^(AR),Meghalaya (ML),Mizoram^a^(MZ), Nagaland^a^(NL), Rajasthan (RJ), Telangana (TS), West Bengal (WB), Andaman and Nicobar Island (AN), Dadra and Nagar Haveli^a^(DN), Lakshadweep^a^(LD), Chhattisgarh (CG), Pondicherry (PY), Daman and Diu (DD)^a^.

^a^These states and union territories do not have medical teaching institutes [7]

### Inputs from the anatomist

Information along with informed consent was sought from the Head, Department of Anatomy of all approved medical institutes of the Union of India. The information regarding difficulties encountered during the acquisition of unclaimed or donated cadavers and suggestions were sought on possible improvements/ modifications in the present Anatomy Acts of their respective states. The information-seeking questionnaire was sent first by speed post, for non-responsive end users it was sent second time along with a self-addressed envelope.

The problem statements of existing Anatomy Acts, highlighting the deficiencies and discrepancies and possible modifications were presented at the National Conference of Anatomical Society of India at Imphal, 2014, and Society of Clinical Anatomist at Amritsar in 2016. The views of eminent anatomists present there were sought and documented.

### Formulation of a model Act

Considering the need for the current scenario of medical education suitable modifications in the Acts are proposed and a model Act has been formulated.

## Results

We could obtain original/amended Anatomy Acts and Bill from eighteen states and Union territories [6–11, 17–27]. Three states confirmed, in response to an RTI query, that there is no Anatomy Acts in their state. Fourteen states and/ Union Territories did not respond to RTI query nor could the authors get Act from internet/stakeholders. In one state (Manipur) the Act was still a bill and was yet to be enacted [24].

Only six states have so far amended their Acts. The years of the amendment are also shown in Table 2.

**Table 2:** Showing the number and the year of amendment/s of various Anatomy Acts

Sr. No	State	Number of amendments	Year/s of amendments
1	DL	1	2014
2	KA	2	1999&2005
3	MH	2	1975 &2000
4	KL	2	1964 & 1988
5	TN	1	1960
6	OD	2	1975 &2012

### The problem statement and comparison of various clauses of the Act is as follows

The sections mentioned are as per the Gujarat Anatomy Act [20]. These sections may differ in serial order in different Acts.

The Act starts with the **Statement of Objects and Reasons**, which states the purpose **(**Table 3**)** for which the Act has been enacted. The purpose does not include ‘the use of donated bodies’ in twelve states namely KL, TN, GA, GJ, HP, HR, PB, CH, SK, UP, MP, and AS [9, 11, 17, 19–23, 25–27].

**Table 3:** Showing differences in the purpose of the Act/s

Sr. no	Purpose of the Act	States
1	Anatomy examination/dissection, research purpose, and other similar purposes.	All available acts of the states.
2	Therapeutic and surgical operations also stated as purpose in addition to point 1	MH,OD, TN,GA,GJ,HR,PB, CH.

### Section: short title, extent, and commencement

This section is uniformly similar across all Acts and contains the year of enactment and amendments thereof.

### Section:definitions

#### Approved institute

All the Anatomy Acts available to us include medical teaching institutes as approved institutes for the purpose of the Act, but the Acts of MH, OD, TN, GA, GJ, HP, HR, PB, CH, MP, and MN also include standalone hospitals (not attached to any medical teaching institute) as approved institute s[6, 10, 11, 14–25].

#### Authorized officer

Authorized officer are the officers competent to Act for the purpose of the Act, especially in cases of handling of an unclaimed body.

In all Acts, officers appointed or authorized to act under this Act shall be deemed to be public servants within section 21 of the Indian Penal Code, 1860 (Central Act 45 of 1860).

#### Unclaimed body

All available Anatomy Acts define the unclaimed body – as the body of a deceased person who has no near relative. Claimants recognized by various Anatomy Acts of the body of the deceased apart from near relatives are shown in Table 4.

**Table 4:** Showing recognized claimants of unclaimed bodies besides near relative

S. no	Recognized Claimants of unclaimed bodies	States
1	Person interested	KA
2	Relatives/public institutes of the religion of the deceased.	KL
3	Servants	UP
4	Personal friends	DL, MN

Acts of several states have included a section for body donation but none of the Act has defined a donor.

### Section: power of state government to authorize the officer to Act under section

#### (section: procedure to handle unclaimed bodies)

In Assam, a notification of the state government clarified that police officer having jurisdiction over the area is the authorized officer [17]. Punjab government notification states that the principals of the government medical colleges are the authorized officers for the purpose of the Act. Whether such notification has been issued by other state governments is not known/available to us.

### Section: unclaimed dead bodies in hospitals, prisons, and public place how to be dealt with

All AnatomyActs have clearly laid down procedures to handle the unclaimed bodies but the procedure differs from state to state.

The states namely HP, HR, PB, CH, SK, and MP only mention the duty of authorized officer on intimation of the unclaimed body that it is to be taken in possession with least practicable delay and handed over to approved institute or be disposed of in the manner prescribed [21–23, 25, 26]. But there is no mention regarding suspicious death. States namely MH, DL, KA, KL, TN, UP, AS, and MN also describe the procedure to be followed, if there is suspicion regarding the cause of death [6, 8, 9, 11, 17, 24, 27, 30]. It is then necessary to send the body for post mortem examination. Gujarat Anatomy Act, 2011 states that on suspicion regarding the cause of death, inquest (police) is to be done subsequent to which the body may be handed over to the approved institute or sent for post mortem examination as per findings of inquest [20].

### Section: doubt or dispute as to near relative to be referred to magistrate of the first class -uniformly similar across all acts

#### Section: donation of bodies or any part thereof deceased persons for anatomical examination etc.

Of the available Acts, eight states namely TN, GA, HP, HR, PB, CH, MP, and AS do not have any provision for the donation of the body [11, 17, 19, 21–23, 25]. Acts of MH, KA, OD, and MN clearly indicate the procedure to be followed in cases when the death of an interested donor occurs at home [6, 8, 10, 24]. This includes the provision of acceptance of death certificate stating the manner and cause of death as per his/her best knowledge by a registered medical practitioner who may or may not have attended the deceased person in the last 7 days. This registered medical practitioner, however, should not be concerned with dealing with body donation.

#### Section: maintenance of records

Except for MH, DL, and GJ, no other state has included maintenance of record in their Acts [6, 7, 20]. Maharashtra Anatomy Act clearly lays down the manner in which the records have to be maintained, specifies the entries and duties of concerned authorities in a time-bound manner [6].

#### Section: refusal to accept unclaimed / donated body

Different states have different clauses with regards to the refusal to accept a donated /unclaimed body by the approved institute as shown in Table 5.

**Table 5:** Summaries the section of refusal to accept donated /unclaimed body

	Name of state	Clause
1	MH, GA, HP, PB, SK, MP, AS	No provision for refusal of unclaimed /donated bodies.
2	KA, KL, TN, UP, MN	It is mentioned that authorised institute shall accept the unclaimed /donated body if required.
3	DL, OD, GJ	Clearly mention the right to refuse an unclaimed /donated body. If senior officer of authorised institute decides that the body is not suitable for educational purposes or the body is not required by the said institute.

#### Section: disposal of the dead body

None of the Acts has included the clause for the disposal of the dead body after its use except for three states i.e. MH, OD, and GJ [6, 10, 20]. While OD and GJ have mentioned the disposal in the prescribed manner, Maharashtra Anatomy Act has dealt with this issue in detail prescribing the manner in which the said disposal should happen as per the religious persuasion of the deceased [6, 10, 20]. The authorized institute is required to transfer the information of disposal to the executive magistrate or any other officers as approved by the State Government within a stipulated time.

#### Section: penalty

All the available Acts include a provision for punishment of a person who obstructs any process laid down in the Act. This punishment varies in different Acts and ranges from penalty of  200 [21, 22, 25] to  5000 [20, 24] and even with provision of imprisonment up to 6 months [10].

**Section: pertaining to duty of police and other officers to assist in obtaining possession of unclaimed bodies**--were similar across all acts

**Section: protection of persons acting under this Act and officers to be public servants**-were similar across all acts

**Section: Act not to prohibit post-mortem examination**

Following states, namely MH, DL, KA, OD, KL, GJ, SK, UP, AS, and MN have a clause stating that nothing in Anatomy Acts prohibits post mortem examination if there is suspicion regarding the cause of death [6–10, 20, 24, 26, 27].

**Section: Power to make rules:** All Acts provide power to the State Government to make rules/amendments.

**Section: transfer and transport of cadavers and anatomical material amongst the authorized institute**

While no Act has a section addressing transfer and transport of cadavers amongst the approved institute. The Government of KA, by gazette notification, has framed the rules and regulations regarding the same in 2005 [31]. Any such notification by any other state government could not be obtained.

The suggestions and views of sixty-six respondents received in response to a questionnaire addressing various sections of the Anatomy Acts are shown in Table 6.

**Table 6:** Overwhelming views and suggestions of the Anatomy fraternity of India regarding various sections/ provisions of Anatomy Acts of their respective states

S. no	Questions Regarding various sections of Anatomy Act of their respective states	Response			VIEWS/SUGGESTIONS OF STAKE HOLDERS
		Yes	No	Nil	
1.	Is approved institution clearly defined?	52	10	2	Although the definition of approved institute implies that it includes all government/private medical and dental institutes. Anatomist want approved institute to be clearly spelt out either in act or in notification.
2.	Is the definition of unclaimed bodies clearly spelt?	47	15	2	
3.	Is procedure to handle unclaimed bodies clearly laid down?	42	20	2	Ways to be devised so that as bodies reach all approved institutes (all government/private medical, dental and allied) unbiased without performing postmortem in specific time frame. Authorized officer should be aware of his responsibilities regarding the act.
4.	Have the responsibilities of authorized officer been clearly defined?	35	27	2	So that the unclaimed body reaches the approved institute before the putrefaction sets in.
5.	Is provision for receiving donated bodies present?	43	18	3	Overwhelming view to include this section in acts lacking it.
6.	Suggestion regarding management of situation, when donated bodies arrive at the approved institute without death certificate.				Should be received only along with police verification report (i.e No Objection certicate-N.O.C.), certificate from village sarpanch or denied in absence of above certificates.
7.	Is transfer or transport of cadaveric materials mentioned?	21	38	5	Should be legally approved.
8.	Does the Act mention the maintenance of records?	28	31	5	Documentation should be mandatory and clearly laid down for bodies or part thereof/bones/anatomic material/still birth fetus. There should be uniformity in the format of body donation forms, acknowledgement letter, maintenance of records of donations and unclaimed bodies etc.
9.	Is procedure of disposal of claims by the “said relatives” clearly laid down?	23	37	4	Should be clearly laid down
10.	Is a clause for penaltyfor thosewho interfere with any procedure of act present?	25	30	9	Should be increased from Rs. 5000/= to Rs. 25,000/=
11.	Is uniformity in Anatomy Acts of various states is need of the day.	53	8	3	Socially and demographically states are different hence not possible. Laws regarding handling of unclaimed body are stringent in certain states which if uniformly applicable will affect the availability of unclaimed body in all states. Only guidelines to be issued by central government.
12.	Any other Suggestions				Officer who should receive the body in approved institute should be clearly defined. Criteria for acceptance or refusal of unclaimed body should be clearly laid down. National body bank registry for uniform distribution of cadavers. Prior informed consent from donor and/or near relative in case of transfer and transport of cadaver.

## Discussion

An Act which is progressive and is based on ethical principals is the need of the hour. While the world is advocating good practices in the acquisition of bodies for anatomical examinations, laws in India must adopt and support practices that are ethical, transparent, and accountable. A progressive step in this direction would be to gradually shift from unclaimed bodies, where no consent of the deceased could be obtained, to donated ones. To inspire confidence in potential body donors transparency in the whole process viz. informed consent of the donor, maintenance of records, disposal of the anatomical material, should be addressed in various clauses of the Act.

Review of available Anatomy Acts highlights glaring discrepancies and deficiencies across various clauses of the Acts. While some states like MH, KA, KL, etc. have amended the Acts with changing times, many states continue with outdated Acts that are not in tune with the changed medical education scenario,socio-cultural fabric, and ethical norms [6, 8, 9].

State reorganization has resulted in smaller states being carved out of larger ones like UK, JH, CG, TS. These states need to adopt the Anatomy Act with a suitable amendment.

As the medical teaching institutes are increasing in number and expanse, they have outreached even the states earlier not having such institutes. Such States /Union Territories also need to enact the Anatomy Act as early as possible (e.g. AN, TR, ML) [32].

The provision of the Anatomy Act is primarily to ethically get cadavers for anatomical examination, dissection, and research, etc. However, the inclusion of surgical operations for therapeutic purposes is out of place especially after the enactment of The Transplantation of Human Organs and Tissues Act,1994 [28] which, under its ambit, includes the use of all human tissues for therapeutic purpose. Hence surgical operations should be deleted from the purpose of the Anatomy Act.

An approved institute as stated in all Acts is a medical teaching institute however inclusion of standalone hospitals not involved in teaching and research is unwarranted and hence should be deleted.

The key person in the enactment of the Anatomy Act is the authorized officer. However, the definition of the authorized officer as per law is very wide and includes all public servants under section 21 of the Indian Penal Code, 1860 (Central Act 45 of 1860). In the absence of government regulations as per who amongst them is the authorized officer for the purpose of this Act creates confusion, thus hampering the proper disposal of duties.

Communication technology has helped in the identification of unclaimed bodies and hence the number of unclaimed bodies is on the decline, but on other hand, our socio-cultural milieu is also changing with times and there are many cases encountered nowadays where the body of a deceased person is not claimed by a near relative. This is more so in the case of elderly individuals staying alone.

While some states have included the provisions of claimants like personal friends, servants, etc. Authors feel that in the absence of near relative the claimants should be a group of persons interested, at least five in number, who should be known and associated with deceased, rather than a single individual. This is important to avoid any legal complications like property disputes.

As was observed a ‘donor’ was not defined even in those Acts which had regulation for body donations. Authors feel that a body donor should be defined as in The Transplantation of Human Organs and Tissues Act, 1994 [28].

Getting consent from the potential donor for the use of his/her body after death is a primary prerequisite for using any human tissue. Hence defining a donor in the Act is very important.

Most of the Acts have a detailed laid down procedure for handling of unclaimed bodies as these were the primary sources of cadavers and still are in many places. After confirmation that the body is unclaimed, the authorized officer has to ascertain the circumstances of death. Whether or not all such unclaimed bodies should be treated as a suspicious death and be subjected to post-mortem examination is answered differently in different Acts. Post-mortem renders the body unsuitable for preservation and hence dries up the source of unclaimed bodies for educational institutes. Large sections of anatomists feel that a police inquest into the cause of death (ruling out foul play) should be sufficient enough and thence the unclaimed body can be handed over to the approved institute [20]. However, it is also acknowledged that an inconclusive police inquest would warrant a post-mortem examination.

Authors note with concern that in India there is a lack of desired sensitivity about the use of unclaimed bodies for dissection and utilising an unclaimed body is generally perceived as a norm. Authors are of strong opinion all stakeholders involved in the acquisition of cadaveric material are sensitised enough so as not treat the cadavers as a mere teaching medium. In this context, use of an unclaimed body, of a person who has not consented voluntarily, is a practice which cannot be justified. Though a clause of the unclaimed body has been retained in the draft Act as a necessary compromise at present, with a feeling of optimism that anatomists and policymakers shall join together to change this situation for better.

The first voluntary body donation of India happened in 1956 when the body of Late Shri Pandurang Sridhar Apte was donated to B. J. Medical College Pune [33]. An increasing trend of awareness towards the noble cause of body donation for education and research is being observed in recent times so much so that proportion of donated bodies have surpassed that of unclaimed ones in some parts of India e.g.Gujarat [34]. Media in India has been of great help in spreading awareness about body donation and publicising donation as an act of philanthropy towards the larger good of society.

Rokade et al. have described various barriers for voluntary body donation such as lack of awareness (as only 22 % of Indians were aware of body donation), spiritual and religious beliefs, apprehension of the body not being treated with dignity and for the right cause [35]. In a progressive Anatomy Act, it is desirable to address these concerns by having appropriate clauses for the ethical handling of the bodies and their proper respectable disposal.

All stakeholders especially Anatomists, have to make a conscious and a concerted effort towards popularising donation by awareness programs, thanksgiving by students, maintaining transparency in the entire process of body donation and taking the help of media, thus enhancing the trust of the community and their participation in process of medical teaching and learning for the larger good of the society [13, 14]. The ultimate aim should gradually change the source of cadavers from unclaimed bodies to donated ones.

**Fig. 1:**
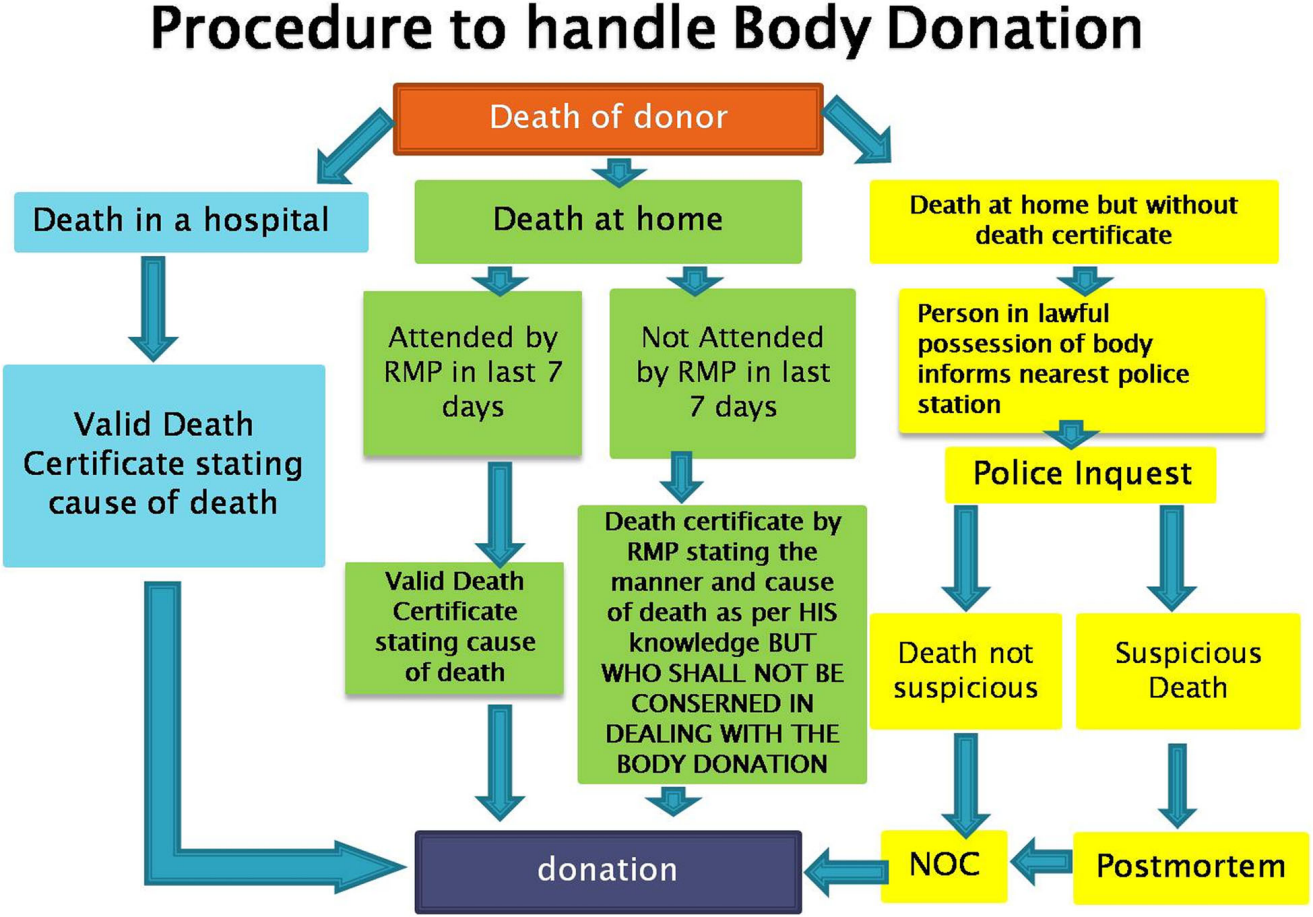
shows the procedure to handle body donation. NOC = No Objection Certificate from the police

This necessitates the inclusion of clause of body donation in all Anatomy Acts. However this trend has also resulted in practical difficulties, legal complications, etc. to obviate such difficulty and facilitate donations which are in legal ambit Fig. 1 explains the suggested procedure to be followed in cases of donation. This has been formulated after combining the provisions in certain Acts e.g. MH, GJ, and views of key users [6, 20].

While the documentation is very important wherever legal processes are involved, Anatomy Acts of only three states i.e. MH, DL, and GJ have prescribed the manner in which the records have to be maintained [6, 7, 20]. The authors feel that this clause should necessarily be included in all Acts.

There are many circumstances where the officer in charge of an approved institute is compelled to refuse a donated or unclaimed body. Such circumstance may be:

- Body unsuitable for embalming/preservation
- Infectious bodies which are hazardous for handlers
- Medico-legal cases
- Lack of infrastructure and resources which limit the proper storage and handling of bodies (cadavers)
- Any other reason as deemed fit by the senior faculty of the Anatomy department.
- This warrants the provision or refusal of the body that should rest with the approved institute.

Increasing awareness about body donation has resulted in increasing queries regarding the disposal of dead bodies after their use; especially by near relatives. To ensure respectable disposal of human bodies and its parts thereof, it must be prescribed by all Acts. Though this is prescribed in Maharashtra Anatomy Act but is least practical and hence needs simplification [6]. Anatomists feel that decent cremation in consecrated ground is both practical and respectful.

Clause for the penalty for ‘whosoever abets the disposal of or obstructs any officer in the execution of Anatomy Act’ is present in all available Acts, however, the penalty varies drastically. Authors support a harsher penalty with the provision of imprisonment as included in the Odisha Anatomy (Amendment) Act, 2012 [10].

There are disparities in the cadavers available for teaching and research across states, between institutes and amongst the government and private running institutes also. While some states and institutes have surplus cadaveric material (sometimes causing difficulty in storage and handling of cadavers) whereas severe dearth of cadavers in some states and institutes is adversely affecting the learning and training of medical students. In absence of any clause for transfer of anatomic material from one approved institute to another, any transport is thus considered illegal as of now. The inclusion of such a clause would ensure equitable distribution, thus ensuring equal standards in medical education. The inclusion of such a clause is an overwhelming demand of almost all Anatomists.

Authors suggest that a nodal centre having a registry of unclaimed or donated cadavers can also aid in ensuring uniform distribution.

The whole exercise of review of Acts and extensive feedback received from end-users culminated in the drafting of a comprehensive model Anatomy Act which has incorporated

i. best of provisions of various Anatomy Acts
ii. has drawn from the global good practices for body donation as adapted to the Indian context
iii. and some modifications and inclusions as suggested by key users

The draft Act (Additional file 1) incorporates the foundation of ethical practices in various clauses viz.

1. Definition of the donor; which encompasses voluntary consent as its essential tenet.
2. While the Act intends to facilitate body donation and move away from the regressive practices like the use of unclaimed bodies, some important clauses added to the Act are

- Procedure to handle body donation is outlined which is both legally tenable and practicable.
- Documentation and record-keeping have been ensured.
- The accountability of stakeholders has been fixed eg. Roles of the authorized officer, approved institute, and dispute resolution have been specified.
- A prime concern of the donor is always the mode of disposal, which has been incorporated in the Act.

## Conclusion

Authors present a draft Anatomy Act addressing the disparities and deficiencies in the existing Anatomy Acts of various States and Union Territories of India and also considering the changing medical education scenario and socio-cultural fabric. This Act ensures the provision of cadaveric material for teaching and training of medical students under the legal ambit. The Act is founded on ethical principles and ensures transparency and accountability for all stakeholders.

It is expected that this Act will ignite a major shift in the attitudes with which human material is used ensuring dignity even after death. It is also expected to guide lawmakers to make suitable amendments to existing legislation and also serve as a reference document for anatomic fraternity across the globe to critically analyze their respective Anatomy Acts.

## Supplementary information

**Additional file 1.**

## Data Availability

The dataset/documents used and/or analyzed during the current study are available from the corresponding author on reasonable request.

## Abbreviations

AN: Andaman and Nicobar Island
AP: Andhra Pradesh
AR: Arunachal Pradesh
AS: Assam
BR: Bihar
CG: Chhattisgarh
CH: Chandigarh
DD: Daman and Diu
DL: Delhi
DN: Dadra and Nagar Haveli
GA: Goa
GJ: Gujarat
HP: Himachal Pradesh
HR: Haryana
IHEC: Institutional Human Ethics Committee
J&K: Jammu and Kashmir
JH: Jharkhand
KA-: Karnataka
KL: Kerala
LD: Lakshadweep
MH: Maharashtra
ML: Meghalaya
MN: Manipur
MP: Madhya Pradesh
MZ: Mizoram
NL: Nagaland
NOC: No Objection Certificate
OD: Odisha
PB: Punjab
PY: Pondicherry
RJ: Rajasthan
RRB: Research and Review board
RTI: Right to Information
SK: Sikkim
TN: Tamil Nadu
TR: Tripura
TS: Telangana
UK: Uttarakhand
UP: Uttar Pradesh
WB: West Bengal
