# Supplementary material for: Changing medical education scenario: a wakeup call for reforms in Anatomy Act
Source: BMC Med Ethics. 2020 Jul 25;21:63. doi: 10.1186/s12910-020-00507-0 (PMC7382862; doi:10.1186/s12910-020-00507-0)
Supplement: Supplementary file 1 — Additional file 1. [file 12910_2020_507_MOESM1_ESM.docx]

**ANNEXURE A**

**THE DRAFT ANATOMY ACT**

**ARRANGEMENT OF SECTIONS**

Statement of Objects and Reasons:

Sections:

1. Short title, extent, and commencement.
2. Definitions.
3. Power of State Government to authorize officers to act under section 4.
4. Unclaimed dead bodies in hospitals, prisons, and public places how to be dealt with.
5. Donation of bodies or any part thereof of deceased persons for anatomical examination etc.
6. Transfer and transport of Cadavers and anatomical material amongst the authorized institutes
7. Maintenance of records
8. Duty of Police and other officers to assist in obtaining possession of unclaimed bodies
9. Refusal to accept the dead body
10. Disposal of the dead body
11. Doubt or dispute as to near relative to be referred to Magistrate of the First Class.
12. Penalty.
13. Officers to be public servants
14. Protection of persons acting under this Act.
15. Act not to prohibit postmortem examination
16. Saving
17. Power to make rules.

* * * *

**STATEMENT OF OBJECTS AND REASONS**

An Act to provide for the supply of unclaimed bodies of deceased or donated bodies or any part thereof of deceased persons to the medical teaching institutions for the purpose of medical education or research including anatomical examinations and dissection and other similar purposes.

1. **Short title, application, and commencement**
2. This Act may be called the Anatomy Act,…..(Year).
3. It applies, in the first instance, to the whole of the States of ………, ………. and ………. and to all the Union territories and it shall also apply to such other State which adopts this Act by a resolution passed in that behalf under clause (1) of Article 252 of the Constitution.
4. It shall come into force in the States of ……, ………, and ……… and in all the Union territories on such date as the Central Government may, by notification, appoint and in any other State which adopts this Act under clause (1) of Article 252 of the Constitution, on the date of such adoption; and any reference in this Act to the commencement of this Act shall, in relation to any State or Union Territory, means the date on which this Act comes into force in such State or Union Territory.

**2. Definitions**

(1) In this Act, unless there is anything repugnant in the subject or context,–

1. “**approved institution”** means a medical, dental, paramedical teaching institution as declared by the State/Central Government by notification in the Official Gazette, to be an approved institution for the purpose of this Act;
2. “**authorized officer**” means an officer authorized under section 3;
3. “**near relative**” means any of the following relatives of the deceased, namely, wife, husband, parent, son, daughter, brother, sister, grandparent, grandson, or granddaughter. and includes any other person who is related to the deceased (i) by lineal consanguinity within three degrees or by collateral consanguinity within six degrees, or (ii) by marriage with any of the relatives aforesaid. The expressions “lineal consanguinity” and “collateral consanguinity” shall have the meaning assigned to them in the Indian Succession Act, 1925, (Central Act XXXIX of 1925) and degrees of relationship shall be computed in the manner laid down in that Act.
4. **“person interested”** means a group of five persons who can be personal friends, servants, relatives/public institutes of the religion of deceased who are interested in the disposal of the body of the deceased person in accordance with the religious usage or social custom of such deceased.
5. **“prescribed”** means prescribed by rules made under this Act;
6. “**unclaimed body**” means the body of a deceased person who has no near relative or whose body has not been claimed by any of his near relatives within such period as may be prescribed.
7. **“donor**” means any person, not less than eighteen years of age, who voluntarily authorizes the donation of his body or a part thereof for the purpose of the Act**.**

**3. Power of State Government to authorize officers to act under section 4.**

The Government may, by notification in the Gazette, authorize for the area to which this Act is applied or for any part thereof, one or more officers to whom a report shall be made under section 4 and who shall be competent to act under the said section.

1. **Unclaimed dead bodies in hospitals, prisons and public places how to be dealt with -**
2. If a person dies in a hospital or in a prison and his body is not claimed by any near relative or person interested in absence of near relative within the prescribed time, the authority in charge of such hospital or prison or any police officer shall, with the least practicable delay report the fact to the authorized officer and the said officer shall take possession of the unclaimed body and except in the case referred to in sub-section (4), hand it over to the authority in charge of a nearest approved institution if it is required by that authority, for the purpose of conducting anatomical examination and dissection or other similar purposes.

Without prejudice to the generality of the provision, the responsibility for immediately reporting the fact to the nearest approved institution and also for arranging the removal of the dead body to the approved institution for preservation from decay, shall be the duty the authorized officer having jurisdiction over the area.

1. If a person dies in any public place in an area in which he had no permanent place of residence and the body of that person is not claimed by any near relative or person interested in absence of near relative within the prescribed time, the authorized officer shall take possession of such unclaimed body and except in the case referred to in sub-section (4), hand it over to the authority in charge of a nearest approved institution if it is required by that authority, for the purposes specified in sub-section (1).
2. Notwithstanding anything contained in sub-sections (1) and (2), the authorized officer shall-

(a)When the approved institution refuses to accept the body for the purpose specified in sub-section(1)

(b) when the deceased person has prior to his death declared that his body shall not be subjected for the purpose of conducting anatomical examination and dissection or other similar purposes.

(c) hand over the dead body to such religious or public institution belonging to the religion of the deceased subject to such conditions as may be prescribed or it shall be disposed of in such manner as may be prescribed.

1. When there is any doubt regarding the cause of death or when for any reason the authorized officer considers it expedient so to do, he shall forward the unclaimed body to the police officer referred to in section 174 of the Code of Criminal Procedure, 1898 (Central Act V of 1898). After the proceedings have been completed the police officer shall deal with the dead body as per subsection (1), (2), and (3).

**5. Donation of bodies or any part thereof of deceased persons for anatomical examination etc.**

(1) If any person at any time before his death had expressed an intention in writing in the presence of two or more witnesses, that his body or any part of his body be given to an approved institution for being used after his death for the purpose of conducting anatomical examination and dissection or other similar purposes, any near relative or person interested in absence of near relative may, unless he has reason to believe that the said intention was subsequently revoked authorize the removal of the dead body or such part thereof to any approved institution for use in accordance with the intention.

(2) Without prejudice to the provisions of sub-section (1) such near relative or person interested in absence of near relative may authorize the removal of the whole body or any part from the body for use for the purposes specified in sub-section (1) unless he has reason to believe -

(a) that the deceased had expressed an objection to his body or any part thereof being so dealt with after his death and had not withdrawn such objection; or

(b) that any near relative of the deceased referred to clause (d) of section 2 objects to the body being so dealt with.

(3) Subject to the provisions of sub-section (4) and (5), the removal and use of the whole body or any part of a body in accordance with an authority given in pursuance of this section shall be lawful and shall be sufficient warrant for the removal of the body or any part thereof and it's use for the purposes of this Act.

(4) In no case shall the body or any part of the body of any person be removed for any of the purposes specified in sub-section (1) from any place where such person may have died

(a )unless a death certificate stating in what manner such person came by his death shall, previously to the removal of the body, has been signed by a registered medical practitioner who attended such person during the illness whereof he died, or, if no such practitioner attended such person during such illness, then by a registered medical practitioner who shall be called in after the death of such person to view his body’, and who shall state the manner and cause of death according to the best of his knowledge and belief, but who shall not be concerned in dealing with the body for any of the purposes aforesaid after removal; and in case of such removal, such certificate shall be delivered together with the body.

(b) Or in absence of the death certificate, the person lawfully in possession of the body shall inform authorized officer/ sarpanch of the village. The authorized officer shall conduct an inquest under section 174 of the Criminal Penal Code subsequent to which he may issue a no-objection certificate to be delivered along with the body of the deceased to the authority in charge of a nearest approved institution receiving the same for any of the purposes aforesaid.

**6. Transfer and transport of Cadavers and anatomical material amongst the authorized institutes**

The dead body received by the approved institute for the purpose of the Act under section 4 and 5 can be transferred and transported to other authorized institutes along with other anatomical materials, provided the deceased/near relative/person interested in absence of near relative have consented. If requested by the said institute on payment of user chargers as decided by senior officer/ body donation committee of the authorized institute but the proper record should be maintained as prescribed.

**7. Maintenance of records**

The authority in charge of an approved institution, on receiving the body of a deceased person for all or any of the purposes of this Act, shall, demand and receive, together with the body, a certificate as aforesaid and shall, after such removal, transmit within 7 days of death to the Executive Magistrate or such officer as may be appointed in this behalf by the Government, a copy of such Certificate and also a return stating on what day and what hour and from whom the body was received, the date and place of death, the sex and (as far as known at the time) the name, age and last place of abode of such person and shall enter the aforementioned particulars in a register to be kept by such authority for that purpose and shall produce such register whenever required to do so by the authorized officer or, as the case may be, by the Executive Magistrate or any officer aforesaid.

The authority in charge of the approved institute shall also maintain a register of dead bodies it used, transported, transferred, and disposed of in such form and such manner as may be prescribed**.**

**8. Duty of Police and other officers to assist in obtaining possession of unclaimed bodies**

All officers and employees of health and other government departments, All officers and servant of a local authority and all village officers and employees, shall be bound to take all reasonable measures to assist the authorities and officers authorized under this Act in the discharge of their duties under this Act.

**9. Refusal to accept the dead body**

The approved institute may refuse to accept an unclaimed or body brought to it under section 5 if a senior officer decides that the body is not suitable for educational purposes or that the body is not required by the said institute. Only intact dead bodies without any type of mutilation can be accepted for embalming and teaching purposes.

However, in case of a body found otherwise fit for donation but not accepted by the Medical Institution, reference will be made to other Medical Institutions who may require it, through a system of networking among the Medical Institutions.

**10. Disposal of the dead body**

Every dead body removed as aforesaid for any of the purposes of this Act, shall before such removal, be placed in a decent coffin or shell or any other thing for holding the dead body, and be removed therein; and that the party removing the same, or causing the same to be removed as aforesaid, shall make provision that such body, after being dealt with for any of the purposes of this Act, be decently cremated or interred in consecrated ground.

**11. Doubt or dispute as to near relative/person interested to be referred to Magistrate of the First Class.**

(1)A magistrate of the First class, for the purpose of deciding any doubt or dispute whether a person is or is not a near relative of a deceased, shall hold a summary inquiry into the matter. Such magistrate will not be required to record the whole of the evidence of a witness, but he shall maintain a memorandum of evidence of a witness and a gist of representations made in the case on the basis of which he may arrive at a decision.

(2) Pending such a decision, the authorized officer shall take all reasonable care and steps to preserve the body of the deceased person from decay.

**12. Penalty**

Whoever disposes of, or abets the disposal of [a dead body] save as permitted by this Act, or obstructs any authority in charge of an approved institution or an authorized officer from handing over, taking possession of, removing or using, such dead body for all or any of the purpose of this Act] shall, on conviction, be punished with fine which

may extend to five thousand Indian Rupees.

**13. Officers to be public servants**

All officers appointed shall be deemed to be public servants within the meaning of section 21 of Indian Penal Code

**14. Protection of persons acting under this Act.**

No suit, prosecution or other legal proceedings shall lie against any person for anything which is in good faith done or intended to be done in pursuance of this Act.

**15. Act not to prohibit postmortem examination**

A. Nothing contained in this Act shall be construed to extend to or to prohibit, any post-mortem examination of any human body required or directed to be made under any law for the time being in force in the State.

B. (1) Nothing in this Act shall be construed as rendering unlawful any dealing with the body or any part thereof of a deceased person which would have been lawful if this Act had not been passed.

( 2 ) Any authority for the removal of the body or any part thereof given in accordance

with the provisions of this Act shall not be deemed to be a contravention of the provisions

of section 297 of the Indian Penal Code].

**16. Power to make rules.**

The …… Government, may, by notification in the Official Gazette, make rules for carrying out the purposes of this Act. All rules made under this section shall be laid for not less than thirty days before the …….. legislature as soon as may be after they are made and shall be subject to rescission by the …….. legislature or to such modification as the ……… the legislature may make during the session in which they are so laid or in the session immediately following.
